# Agrp neuron activity is required for alcohol-induced overeating

**DOI:** 10.1038/ncomms14014

**Published:** 2017-01-10

**Authors:** Sarah Cains, Craig Blomeley, Mihaly Kollo, Romeo Rácz, Denis Burdakov

**Affiliations:** 1 The Francis Crick Institute, Mill Hill Laboratory, London NW7 1AA, UK; 2 Department of Neuroscience, Physiology and Pharmacology, Division of Biosciences, University College London, London WC1E 6BT, UK; 3 Institute of Psychiatry, Psychology and Neuroscience, Department of Developmental Neurobiology, King's College London, London WC2R 2LS, UK

## Abstract

Alcohol intake associates with overeating in humans. This overeating is a clinical concern, but its causes are puzzling, because alcohol (ethanol) is a calorie-dense nutrient, and calorie intake usually suppresses brain appetite signals. The biological factors necessary for ethanol-induced overeating remain unclear, and societal causes have been proposed. Here we show that core elements of the brain's feeding circuits—the hypothalamic Agrp neurons that are normally activated by starvation and evoke intense hunger—display electrical and biochemical hyperactivity on exposure to dietary doses of ethanol in brain slices. Furthermore, by circuit-specific chemogenetic interference *in vivo*, we find that the Agrp cell activity is essential for ethanol-induced overeating in the absence of societal factors, in single-housed mice. These data reveal how a widely consumed nutrient can paradoxically sustain brain starvation signals, and identify a biological factor required for appetite evoked by alcohol.

## Abstract (web summary)

It is well known that alcohol consumption leads to overeating however the neural mechanisms are unclear. Here the authors demonstrate that hunger promoting Agrp neurons in hypothalamus are also activated by ethanol and are necessary for ethanol-induced overeating.

Drinking an *apéritif* to stimulate appetite has been mentioned as a human practice since at least the 5th century AD^1^. Modern studies confirm that alcohol intake acutely stimulates eating, and correlates with obesity^2^,^3^,^4^,^5^,^6^. Due to the rising incidence of obesity and its co-morbidities, the link between alcohol intake and overeating is becoming a recognized clinical concern^2^,^3^,^4^. Popular explanations for alcohol-induced overeating include an alcohol-induced loss of self-control, leading to a disregard for societal constraints on eating^4^,^6^,^7^. Biological factors required for alcohol-induced overeating remain elusive^2^,^3^,^4^. In homoeostatic models of eating control, the brain's appetite-stimulating signals are negatively regulated by nutrients^8^,^9^,^10^,^11^. An essential eating-stimulating signal is provided by Agrp neurons, a starvation-activated, molecularly distinct hypothalamic cell type^12^,^13^. The Agrp cells are found in mice and humans, and their chemogenetic or optogenetic stimulation is sufficient to cause rapid overeating, even in the absence of energy shortage^14^,^15^,^16^,^17^. Such eating despite energy sufficiency resembles the nutritionally paradoxical nature of alcohol-induced overeating. Although ethanol (EtOH, the active ingredient in alcoholic beverages) is the second most calorie-dense nutrient after fat, it stimulates rather than inhibits eating^2^,^3^,^4^. This suggests that EtOH creates a hunger state, but does not resolve whether EtOH highjacks normal homoeostatic signals of starvation, or evokes a different (for example, hedonic) hunger. Causal links between EtOH-induced overeating and brain hunger signals remain elusive. Establishing such a link would require a demonstration that EtOH-induced overeating is prevented by a selective interference with a molecularly defined hunger pathway.

Most molecular pathways associated with human obesity reside in the brain^18^, and are conserved in lower animals. Therefore, we hypothesized that EtOH may stimulate eating across species by distorting brain hunger signals. Here we probed this by: (1) determining EtOH impact on natural eating of animals isolated from social pressures, (2) examining EtOH action on genetically defined hunger signals and (3) establishing causality between specific hunger signals and EtOH-induced overeating. We present results suggesting that, in mice, EtOH sustains the activity of eating-stimulating hypothalamic Agrp cells, and Agrp cell activity is required for eating stimulation by EtOH.

## Results

### Effect of EtOH on food intake in mice

**Figure 1:**
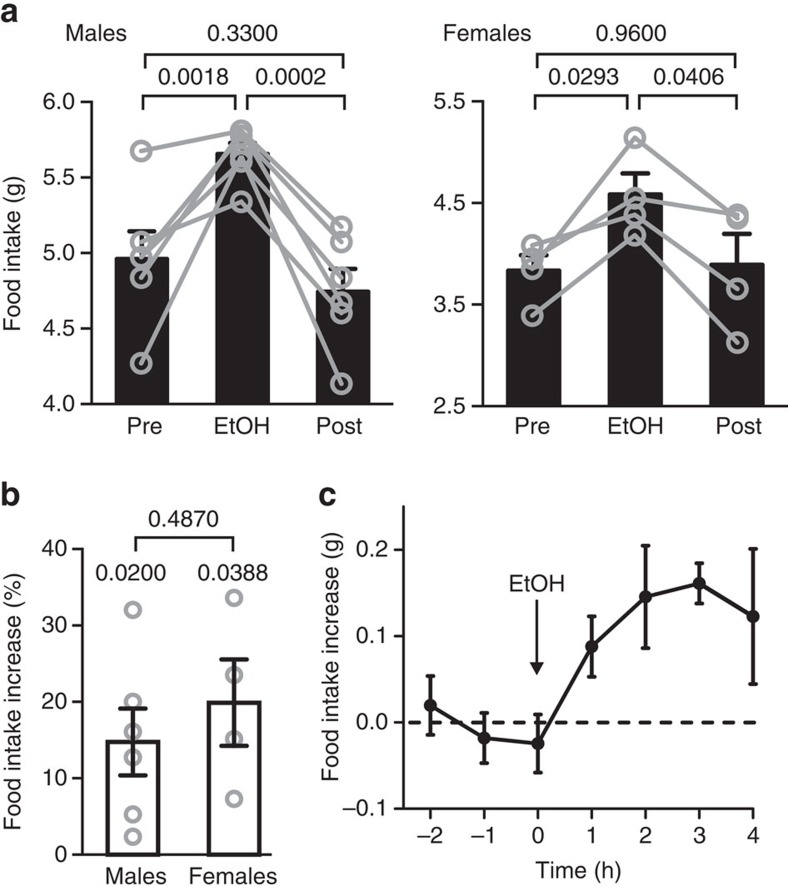
Effect of EtOH on feeding in mice. (**a**) Effect of EtOH on daily food intake (one 2 g kg^−1^ i.p. EtOH injection per day for 3 days; see Methods). Values are averages per mouse per day. Pre=3 days before EtOH, post=3 days after ethanol (mice i.p. injected with saline on non-EtOH days). One-way repeated measures analysis of variance: males, treatment *F*(2, 10)=21.82, *P*=0.0002, *n*=6 mice; females, treatment *F*(2, 6)=7.628, *P*=0.0225, *n*=4 mice. Numbers between bars are *P* values from Tuckey's multiple comparison corrections. Bars and error brackets are means±s.e.m. (**b**) EtOH-induced food intake in males versus females from experiment in A. Two-tailed unpaired *t*-test, *t*=0.7286, d.f.=8, *P* value indicated between bars. Numbers above bars are *P* values from one-sample *t*-tests: males, *t*=3.3672, d.f.=5; females, *t*=3.5242, d.f.=3. Bars and error brackets are means±s.e.m. (**c**) Food intake induced by EtOH injection (arrowed, 2 g kg^−1^), shown at hourly time points (*n*=4 male mice, ‘food intake increase' was calculated by subtracting food intake after saline injection from food intake after EtOH injection, for each mouse and time point). Values are means±s.e.m.

First, to test if EtOH-induced overeating occurs in non-human mammals, we examined the effects of EtOH on food intake of mice. We used single-housed mice to minimize social confounders, male and female mice to examine gender differences, and intraperitoneal (i.p.) administration of EtOH for precise dosage and to remove taste confounders. In an ‘alcoholic weekend' experiment, each animal was given EtOH for three days, and saline for 3 days before and after (see Methods for consideration of EtOH dosage). The food intake was significantly and reversibly increased on the EtOH days, and the magnitude of this increase was similar in males and females (Fig. 1a,b; statistics are given in the figure legends). The effect of EtOH on food intake was also apparent at finer (hourly) time points (Fig. 1c; Supplementary Fig. 2). These data suggest that alcohol-induced overeating is an evolutionarily conserved biological phenomenon occurring across mammals, irrespective of aesthetic beliefs and social conditioning.

### Effect of EtOH on hypothalamic Agrp cells

**Figure 2:**
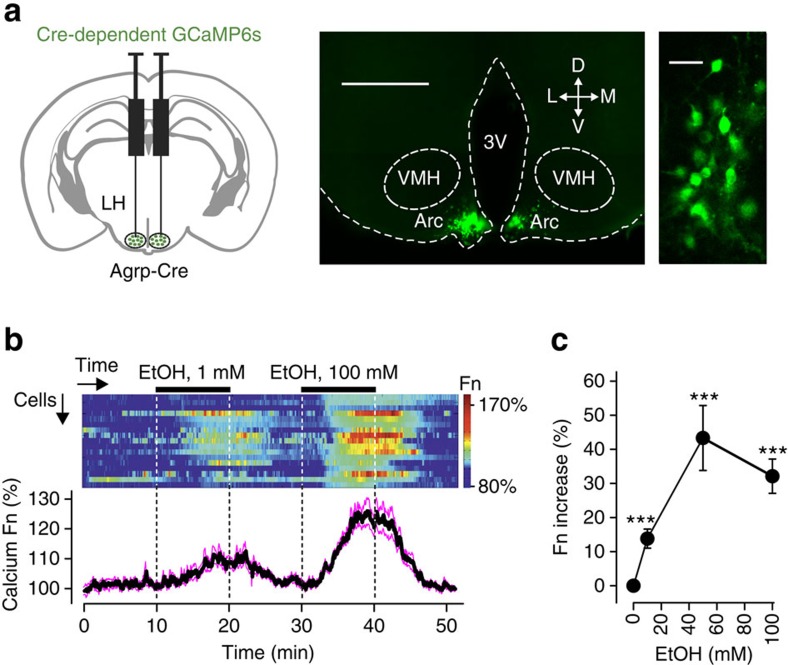
Effects of EtOH on cytosolic biochemical signals of Agrp neurons. (**a**) Left, targeting scheme for the GCaMP6s. Centre, localization of GCaMP6s. 3V, third ventricle; Arc, arcuate nucleus; D, dorsal; L, lateral; LH, lateral hypothalamus; M, medial; VMH, ventromedial hypothalamus, V, ventral. Scale bar, 500 μm. Right, GCaMP6s in Agrp cells at higher zoom. Representative example from 10 brains. Scale bar, 30 μm. (**b**) GCaMP6s response of Agrp cells (*n*=17) to EtOH (top), corresponding means±s.e.m. (bottom). (**c**) Relation between EtOH concentration and peak GCaMP6s response of Agrp cells (from experiments such as shown in **b**), *n*=12–17 cells for each concentration. One-sample *t*-tests: 1 mM, *t*=9.727, d.f.=16; 50 mM, *t*=8.794, d.f.=11; 100 mM, *t*=12.64, d.f.=15; ***P<0.0001.

Second, we sought to establish whether EtOH rapidly modulates endogenous hunger signals. To test if Agrp cell activity is modulated by EtOH, we injected a Cre-inducible ultrasensitive calcium activity reporter GCaMP6s (ref. 19) into the hypothalamic arcuate nucleus of Agrp-Cre mice (Fig. 2a; Methods). Subsequent imaging of the Agrp-GCaMP6s network in brains slices revealed rapid, dose-dependent and reversible EtOH-induced activation of Agrp neurons (Fig. 2b,c). After i.p. injection of EtOH concentrations similar to those used in our *in vivo* experiments, deep brain EtOH concentrations have been measured to reach 30–80 mM in rodents^20^,^21^,^22^,^23^ (see ‘Ethanol dosage' in Methods for further discussion). We found that such EtOH concentrations produced similar Agrp cell activation (≈40% cytosolic calcium elevation, Fig. 2c) to that caused by fasting or physiological hunger hormones^24^.

**Figure 3:**
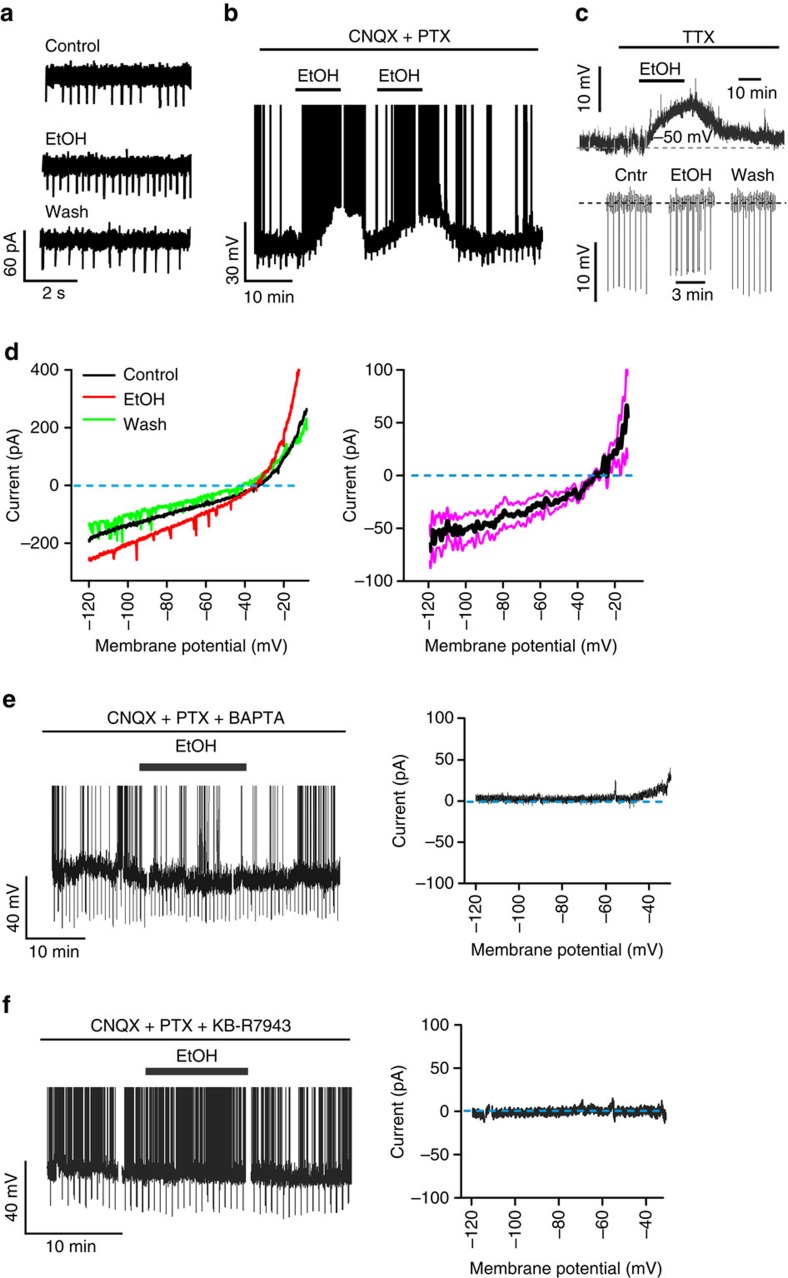
Effects of EtOH on membrane electrical properties of Agrp neurons. (**a**) Effect of 50 mM EtOH on spike rate in cell-attached recordings. Representative example of six cells. Firing before EtOH=1.85±0.35 Hz, after=3.72±0.52 Hz, paired *t*-test: *t*=4.613, d.f.=5, *P*=0.0058. (**b**) Effect of 50 mM EtOH on membrane potential, under synaptic blockade with of 10 μM CNQX and 50 μM PTX. Representative example of eight cells, spikes are truncated at +20 mV. Firing before EtOH 1.07±0.22 Hz, after=7.18±1.58 Hz, paired *t*-test: *t*=3.757, d.f.=7, *P*=0.0071. (**c**) Effect of 50 mM of EtOH on membrane potential in whole-cell recordings, under synaptic blockade with 1 μM TTX. *Top*, membrane potential response; Bottom, responses to hyperpolarizing current injections (resting potentials artificially aligned to dotted line to facilitate comparison). Representative example of eight cells. Mean depolarization was 9.38±0.82 mV, one-sample *t*-test: *t*=11.4, d.f.=7, *P*<0.0001. (**d**) Effect of 50 mM EtOH on whole-cell current–voltage relation. Left, membrane voltage ramps (representative example of 5 cells). Right, net EtOH-induced current (means±s.e.m., *n*=5 cells). (**e**) Effect of 50 mM EtOH on membrane potential (left, spikes are truncated at 0 mV), and net EtOH-induced current (right), in the presence of 10 mM intracellular BAPTA. Representative examples of nine cells. Firing before EtOH=0.96±0.21 Hz, after=0.92±0.20 Hz, paired *t*-test: *t*=1.110, d.f.=8, *P*=0.2994. (**f**) Effect of 50 mM EtOH on membrane potential (left, spikes are truncated at 0 mV), and net EtOH-induced current (right), in the presence of 70 μM KB-R7943. Representative example of *n*=8 cells. Firing before EtOH=0.64±0.19 Hz, after=0.62±0.17 Hz, paired *t*-test: *t*=0.4760, d.f.=7, *P*=0.6485.

The calcium liberation implies activation of biochemical cascades in neurons^25^, but does not directly prove that the electrical spiking of Agrp neurons, the signal driving eating^16^, is increased. Thus, we also examined the electrical activity and membrane currents of Agrp cells in brain slices. In cell-attached electrical recordings (Methods), EtOH acutely and reversibly accelerated Agrp cell spiking (Fig. 3a). Similar effects were seen in whole-cell membrane potential recordings under pharmacological synaptic isolation (Fig. 3b,c), consistent with a direct postsynaptic action of EtOH. In contrast, non-Agrp arcuate neurons (that is, putative satiety cells^16^) were not excited by EtOH (Supplementary Fig. 1A). While this suggests a degree of cell-type selectivity of EtOH actions in the arcuate, we note that the unidirectional inhibition of satiety cells by hunger cells^26^,^27^ implies that such selectivity may not be critical for increasing eating (Discussion).

We next addressed mechanisms of the EtOH-induced excitation. Membrane potential recordings revealed that EtOH-induced excitation involves membrane depolarization and increased conductance (Fig. 3b,c). Whole-cell current–voltage relations further showed that this conductance increase is due to an EtOH-activated non-selective ionic current (Fig. 3d, equilibrium potential=−32±3.1 mV; *n*=7 cells). Buffering cytosolic Ca^2+^ increases by 10 mM BAPTA in the pipette solution abolished EtOH-induced excitatory actions (Fig. 3e), suggesting that a rise in cytosolic calcium is required for EtOH action. Cytosolic calcium elevations can activate plasmalemmal 3Na^+^/Ca^2+^ exchanger (NCX), which can trigger membrane depolarization and excitation due to NCX electrogenicity^28^,^29^,^30^,^31^,^32^. Thus, we tested whether EtOH-induced excitation of Agrp neurons requires this transporter. Bath application of the selective NCX inhibitor KB-R7943 (refs 31, 33) prevented EtOH-induced excitation (Fig. 3f). Together, these data suggest that EtOH-induced excitation of Agrp cell membrane is mediated by cytosolic calcium signals and plasmalemmal NCX activity. Overall, these data demonstrate that EtOH evokes a functional remodelling in the brain's biophysical generators of hunger drive, thereby sustaining false ‘starvation alarms' despite extracellular nutrient sufficiency.

### Effect of Agrp cell silencing on EtOH-induced eating

**Figure 4:**
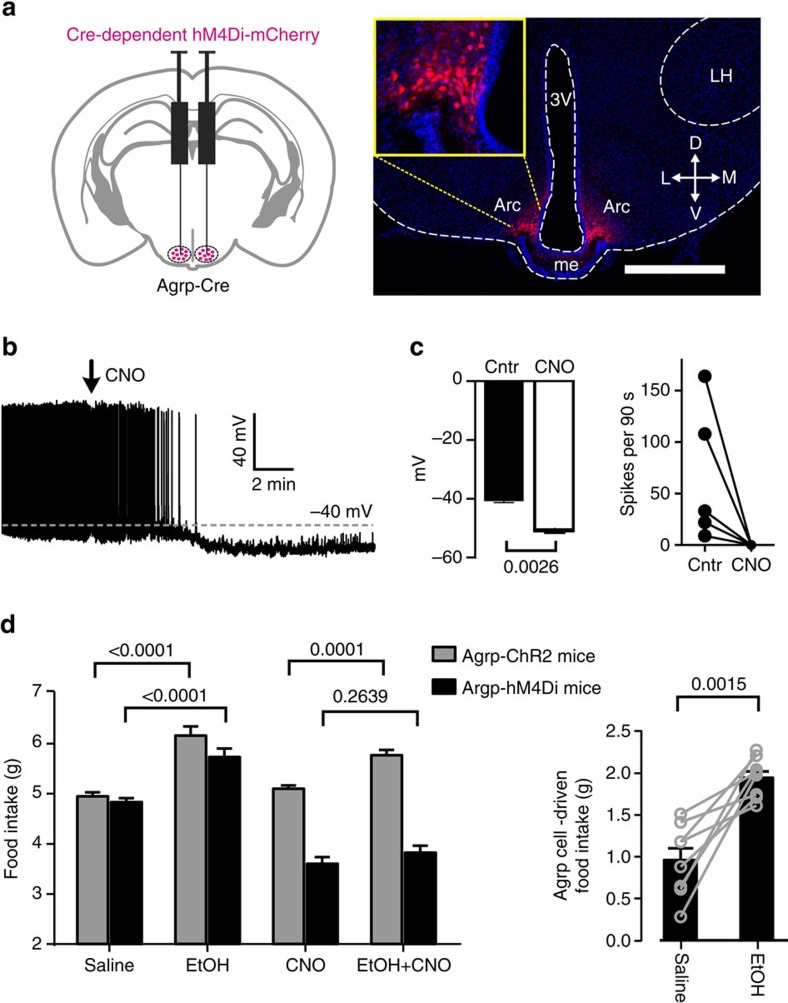
EtOH-induced overeating requires Agrp neuron activity. (**a**) Left, targeting scheme for hM4Di-mCherry. Right, localization of hM4Di-mCherry. 3V, third ventricle; Arc, arcuate nucleus; D, dorsal; L, lateral; LH, lateral hypothalamus; M, medial; V: ventral. Inset, hM4Di-mCherry in Agrp cells at higher zoom. Scale bar, 500 μm. Representative example from four brains. (**b**) Effect of 5μM CNO on Agrp-hM4Di cell firing. Representative example of five cells. (**c**) Left, group data of membrane potential effect in experiment shown in **b** (*n*=5 cells). *P* value is from a paired *t*-test, *t*=6.689, d.f.=4. Right, Spike firing responses in experiment shown in **b** for all cells. (**d**) Left, effects of EtOH (2 g kg^−1^ i.p.) and CNO (5 mg kg^−1^ i.p.) on food intake of Agrp-Cre mice expressing hM4Di or ChR2 in Agrp cells. Food intake under each drug condition represents a mean of 3 days of drug treatment (same design as in Fig. 1a). Four male mice per group (age/gender-matched littermate pairs). Two-way repeated measures analysis of variance: treatment *F*(3, 18)=129.9, *P*<0.0001; genotype *F*(1, 6)=45.44, *P*=0.0005; interaction *F*(3, 18)=53.28, *P*<0.0001; numbers between bars are *P* values from Tuckey's multiple comparison corrections. Right, Agrp cell-driven food intake (defined as reduction in food intake evoked by CNO in Agrp-hM4Di mice) in the presence and absence of EtOH (*n*=8 mice). Number above bars is *P* value from a two-tailed paired *t*-test, *t*=5.055, d.f.=7. Values shown are individual points and/or means±s.e.m.

Third, to investigate whether Agrp cell activity is required for EtOH-induced overeating, we targeted Cre-dependent neuroinhibitory receptor hM4Di, or control protein ChR2-mCherry, to Agrp neurons in Agrp-Cre mice (Fig. 4a; Supplementary Fig. 1B). This made it possible to block Agrp-hM4Di neuron spiking by the physiologically inert hM4Di receptor ligand CNO (Fig. 4b,c). The CNO concentrations we used did not affect spiking of Agrp-ChR2 neurons (Supplementary Fig. 1B), nor eating in mice not expressing hM4Di (Supplementary Fig. 1C,D), confirming that CNO did not modulate the parameters under study without hM4Di. In the absence of CNO, EtOH increased food intake in mice with Agrp-hM4Di or Agrp-ChR2 neurons, (Fig. 4d), confirming that transgene expression *per se* does not prevent ethanol-associated eating. We then gave all mice CNO, to suppress Agrp neurons of Agrp-hM4Di mice. Under this condition, eating of Agrp-hM4Di mice is reduced (Fig. 4d), as previously reported^17^, but—importantly—despite this lower baseline, eating stimulation remains possible by eating-control neurons downstream of Agrp^34^. However, despite this existence of neurons that can increase eating in the absence of Agrp^34^,^35^, we found that in the presence of CNO, EtOH no longer stimulated food intake of Agrp-hM4Di mice (but stimulated it normally in control Agrp-ChR2 mice, Fig. 4d, left plot). While these findings do not preclude EtOH actions on targets other than Agrp, they do imply that such actions are insufficient to cause eating without Agrp cell activity.

Finally, we analysed whether EtOH controls the contribution of the natural activity of Agrp cells to eating *in vivo*. We hypothesized that if EtOH-activated Agrp neurons *in vivo* (as predicted by *in vitro* data, Figs 2 and 3), then EtOH should increase Agrp cell-dependent eating. Because CNO silences Agrp-hM4Di cells (Fig. 4b,c), the magnitude of the CNO effect on eating in Agrp-hM4Di mice reflects Agrp cell-mediated eating, that is, *in vivo* Agrp cell activity (if Agrp cell activity is low, its silencing will have a small effect on eating, and if Agrp cell activity is high, its silencing would have a large effect). We found that the effects of CNO on eating in Agrp-hM4Di mice were significantly greater in the presence of EtOH (Fig. 4d, right plot), consistent with the idea that EtOH increases Agrp cell contribution to eating *in vivo* (Discussion).

Together, these data identify Agrp cell activity as a critical process required for the EtOH action on food intake.

## Discussion

Agrp cell activity potently increases motivation for feeding, and has been called a neural correlate of hunger^17^. Thus our data suggests that alcohol sustains fundamental appetite signals, not just disinhibits their behavioural manifestation. Apart from the Agrp cells, other powerful controllers of food intake exist in the brain, for example, MC4R neurons of the paraventricular hypothalamus^36^, Vgat neurons of the lateral hypothalamus^37^ and neurons of the parabrachial nucleus^34^. Crucially, the control of feeding by some of these modules does not require Agrp neurons^35^. Therefore, alcohol actions on the latter non-Agrp eating controllers would be expected to alter eating even when Agrp cells are silenced. However, we found that when Agrp cells were chemogenetically silenced, alcohol no loger modulated food intake. Thus, we propose that the alcohol-associated activity of Agrp neurons—mediated either by direct alcohol actions described here, or via a still-unknown upstream element—is the critical step in alcohol-induced overeating.

There are previous reports of ethanol actions on diverse cellular targets, including eating-regulating systems such as midbrain dopamine neurons, and on diverse and ubiquitously expressed molecular targets, such as neuronal GABA and NMDA receptors^38^,^39^,^40^,^41^,^42^. We believe that these previous reports are compatible with our findings and conclusions. First, our finding that the Agrp cells are an essential locus of alcohol-induced eating does rule out that there are other sites in the brain, where alcohol can plausibly influence eating. Second, similar to previous studies of other neurons, we found that ethanol acts on Agrp neurons by modulating fairly ubiquitous molecular operators (calcium and NCX), rather than a process that is unique to Agrp cells. We propose that this lack of specificity at the cellular and molecular levels is compatible with a specific action at the whole-system level, due to the non-random, directed interactions between different neuronal controllers of behaviour^43^. Non-random neuronal connectivity has been reported throughout the brain^43^, including appetite systems (for example, Agrp cells inhibit POMC cells but not vice versa^27^). Such a unidirectional network connectivity would ‘filter' non-selective actions at the cellular/molecular levels (for example, direct ethanol-induced excitation of both hunger and satiety cells) into a behaviour-selective action (that is, eating versus non-eating) at the system output level. Testing this proposal by developing tools for disrupting directed connectivity, in conjunction with cell-type specific *in vivo* recordings, is an interesting direction for future work, especially considering that Agrp neurons can be modulated differently *in vitro* and *in vivo*.

Overall, our findings provide an explanation for how a commonly consumed nutrient may generate a positive feedback on energy intake, offering new mechanistic and conceptual insights into pathological overeating behaviour linked to morbid disorders.

## Methods

### Animals and feeding studies

All animal procedures followed United Kingdom regulations (Animals, Scientific Procedures Act 1986) and were approved by local welfare committees and veterinarians. Adult (10–12-week old) male and female mice on the C57/BL6 background were used for all experiments.

For experiments involving transgene expression in Agrp neurons, we used, where indicated, two previously described and characterized transgenic mouse lines: Agrp-IRES-Cre (Jax no. 012899 (ref. 44)) and NPY-hrGFP (Jax no. 006417 (ref. 45)). Mice were kept on a standard 12 h light–dark cycle (lights on at 7:00), and on standard mouse chow (LabDiet 5021) and water *ad libitum*. Room temperature was kept at 22 °C. During food intake measurements, the chow was provided in ‘anti-spill' cylindrical hoppers (TSE Systems). In Fig. 1a,b,d, measurements were performed in home cages by lowering the hoppers onto cage floor through a modified cage lid, and food intake was measured daily by manual weighing of the hoppers. In Fig. 1c, food was weighed automatically in suspended-hopper cages (TSE PhenoMaster) equipped with custom-elevated floors (with openings to maintain hopper suspension) that brought food closer to the floor to facilitate access despite potential ethanol-induced motor incoordination^46^. When placed in unfamiliar cages, mice were allowed to acclimatize for at least one week before measurements.

Drugs (EtOH 2 g kg^−1^, CNO 5 mg kg^−1^) were sterile-filtered and injected i.p. at 12.65 μl g^−1^ total volume (0.9% NaCl saline was used as vehicle). Control i.p. injections contained saline only (12.65 μl g^−1^). Mice were injected at 19:00 (the beginning of the dark phase) and subsequent eating was measured by weighing the food consumed at different times after injection. In an alternative experimental design (which produced similar data to the main findings), mice received a single 1 g kg^−1^ EtOH i.p. injection at 16:00 and food intake was monitored using standard TSE Phenomaster cages (Supplementary Fig. 2).

### Ethanol dosage

For *in vivo* experiments, we used EtOH doses of 2 g kg^−1^ in Figs 1 and 4, and 1 g kg^−1^ in Supplementary Fig. 2; for *in vitro* experiments, we used 10–100 mM EtOH. These doses were based on the following considerations. First, the i.p. EtOH doses we used are compatible with studies of mouse behaviour^47^, and have been reported to elevate deep brain EtOH concentrations to 20–80 mM in rodents *in vivo*^20^,^21^,^22^,^23^, which we here mimicked in brain slices. We confirmed these reports of deep brain ethanol elevations using a custom EtOH sensor in the arcuate nucleus (Supplementary Fig. 3).

Second, these doses resemble those relevant for humans (after correcting for body weight). Specifically, these doses would approximately represent the amounts reported to be consumed by 27% of young UK people at least once per week (>18 units alcohol, according to a UK government survey for 2012: http://www.ons.gov.uk/ons/rel/ghs/opinions-and-lifestyle-survey/drinking-habits-amongst-adults--2012/sty-alcohol-consumption.html). Assuming that 1 alcohol unit contains 8 g EtOH, the 18 units consumed by a 70 kg human equates to an ethanol dose of 2.06 g kg^−1^. The blood EtOH concentration of a 70 kg human male was estimated (Widmark formula) to be ∼50 mM after consuming the 18 units of alcohol. Since EtOH readily crosses membranes and rapidly equilibrates across the blood–brain barrier, similar EtOH concentrations occur in the blood and the brain, and in different brain regions^20^,^23^.

### Network activity imaging

For calcium imaging of Agrp cell activity, AAVs carrying Cre-dependent GCamp6s (rAAV9.CAG.Flex.GCaMP6s.WPRE.SV40; titre 2.74 × 10^13^ gc ml^−1^, Penn Vector Core) were stereotaxically injected into the arcuate nucleus of the Agrp-Cre mice. Two 100 nl injections of the GCaMP6s virus were made at: −1.4 mm from Bregma; ±0.3 mm from midline; and −5.80 and −5.70 mm from skull surface. Cre dependency of viral expression was confirmed by injections of the Cre-dependent AAV into the brains of Cre-negative mice (*n*=3 mice). Acute coronal brain slices were prepared 10–14 days after AAV injection, and confocal imaging and analysis of GCaMP6s signals was performed as previously described^48^.

### Single-neuron electrophysiology

Acute coronal brain slices were prepared and cell-attached and whole-cell recordings performed as previously described^49^. Briefly, fluorescent neurons were visualized an upright Olympus BX51WI microscope equipped with an oblique condenser, a xenon lamp and appropriate filter sets. Somatic recordings were carried out at 37 °C using a HEKA EPC 10 USB patch-clamp amplifier controlled by Patchmaster software (HEKA Elektronik, Germany), after slices were allowed to equilibrate in the recording chamber for 10–15 min. Arcuate Agrp neurons were identified for slice electrical recordings using either Cre-dependent fluorescent transgenes in Agrp-Cre mice (hD4Di-mCherry, ChR2-mCherry); or *NPY-hrGFP* mice since all NPY expression in arcuate neurons co-localizes with Agrp^50^.

### Chemogenetics

To target the CNO-activated inhibitory receptor hM4Di to Agrp cells^17^,^51^, Cre-dependent AAV (rAAV8/hSyn-DIO-hM4D(Gi)-mCherry; titre 5.30 × 10^12^ gc ml^−1^, Penn Vector Core) was stereotaxically injected into the arcuate hypothalamus of the Agrp-Cre transgenic mice. Two 100 nl injections of rAAV8/hSyn-DIO-hm4(Gi)-mCherry or a control Cre-dependent protein (AAV2/EF1a-DIO-ChR2(E123T/T159C)-mCherry, 7.3 × 10^12^ gc ml^−1^, UNC Vector Core) were made at: −1.4 mm from bregma; ±0.3 mm from midline; and −5.80 and −5.70 mm from skull surface. Agrp-Cre mice were first injected with saline during the first 3 days (12.65 μl g^−1^ body weight, i.p.) at dark onset. They were then supplied with a single dose of CNO (5 mg kg^−1^ body weight in saline i.p.) at dark onset during the next 3 days.

### Drugs and solutions

EtOH (absolute analytical reagent grade) was from Fisher Scientific. CNO and KB-R7943 were from Tocris Bioscience. For synaptic isolation, we used CNQX 10 μM, TTX 1μM, and D-AP5 50 μM (Sigma Aldrich UK) and PTX 50 μM (Tocris Bioscience). All other drugs were from Sigma. Artificial cerebrospinal fluid was gassed continuously with 95% O_2_, 5% CO_2_ and contained the following (in mM) 125 NaCl, 2.5 KCl, 1 MgCl_2,_ 2 CaCl_2,_ 1.2 NaH_2_PO_4_, 21 NaHCO_3_, 1 D-(+)-glucose. For whole-cell recordings, patch-clamp pipettes were filled with intracellular solution containing the following (in mM): 120 K-Gluc, 10 KCl, 10 HEPES, 0.1 EGTA, 4 K_2_ATP, 2 Na_2_ATP, 0.3 Na_2_GTP, 2MgCl2, pH 7.3 with KOH.

In Fig. 3, KB-R7943 was applied extracellularly and 10 mM BAPTA was applied intracellularly, as in ref. 32.

### Immunohistochemistry

To confirm hM4Di-mCherry transgene targeting to the arcuate nucleus, mice were perfused with 4% paraformaldehyde, and dissected brains were cryoprotected in 30% sucrose solution for a minimum of 48 h. Free floating cryosections (50 μM) of the arcuate nucleus and cell nuclei were counterstained with Hoescht.

### Statistical analyses

Statistical tests and descriptive statistics were performed as described in the figure legends. In general, we found that 20–30% of Agrp neurons did not respond to EtOH application in our *in vitro* preparation (for example, see Fig. 2b); these non-responding cells were included in group analysis of calcium responses to assess net effect of EtOH on all Agrp neurons (Fig. 2c), but excluded from group quantification of electrical responses to measure EtOH response size at the level of individual cells (Fig. 3a–d). In parametric tests, normality was assessed with Shapiro–Wilk, Lilliefors, Anderson–Darling and Kolmogorov-Smirnov (KS) tests, and variances were assessed for homogeneity using Levene's, Brown–Forsyth and *F*-tests. The analysis was performed using Origin 2015 (OriginLab) or GraphPad Prism 6.

### Data availability

The data that support the findings of this study are available from the authors on reasonable request.

## Additional information

**How to cite this article:** Cains, S. *et al*. Agrp neuron activity is required for alcohol-induced overeating. *Nat. Commun.* **8,** 14014 doi: 10.1038/ncomms14014 (2017).

**Publisher's note:** Springer Nature remains neutral with regard to jurisdictional claims in published maps and institutional affiliations.

## Supplementary Material

Supplementary Information Supplementary Figures
