# Corrigendum: Agrp neuron activity is required for alcohol-induced overeating

**DOI:** 10.1038/ncomms15668

**Published:** 2017-05-19

**Authors:** Sarah Cains, Craig Blomeley, Mihaly Kollo, Romeo Rácz, Denis Burdakov

Nature Communications 8: Article number: 14014; DOI: 10.1038/ncomms14014 (2017); Published 01 10 2017; Updated 05 19 2017

The original version of this Article contained an error in the email address of the corresponding author Sarah Cains. The correct email is cains@hotmail.co.uk. The error has been corrected in the HTML and PDF versions of the Article.

