# Supplementary material for: Agrp neuron activity is required for alcohol-induced overeating
Source: Nat Commun. 2017 Jan 10;8:14014. doi: 10.1038/ncomms14014 (PMC5234092; doi:10.1038/ncomms14014)
Supplement: Supplementary Information — Supplementary Figures [file ncomms14014-s1.pdf]

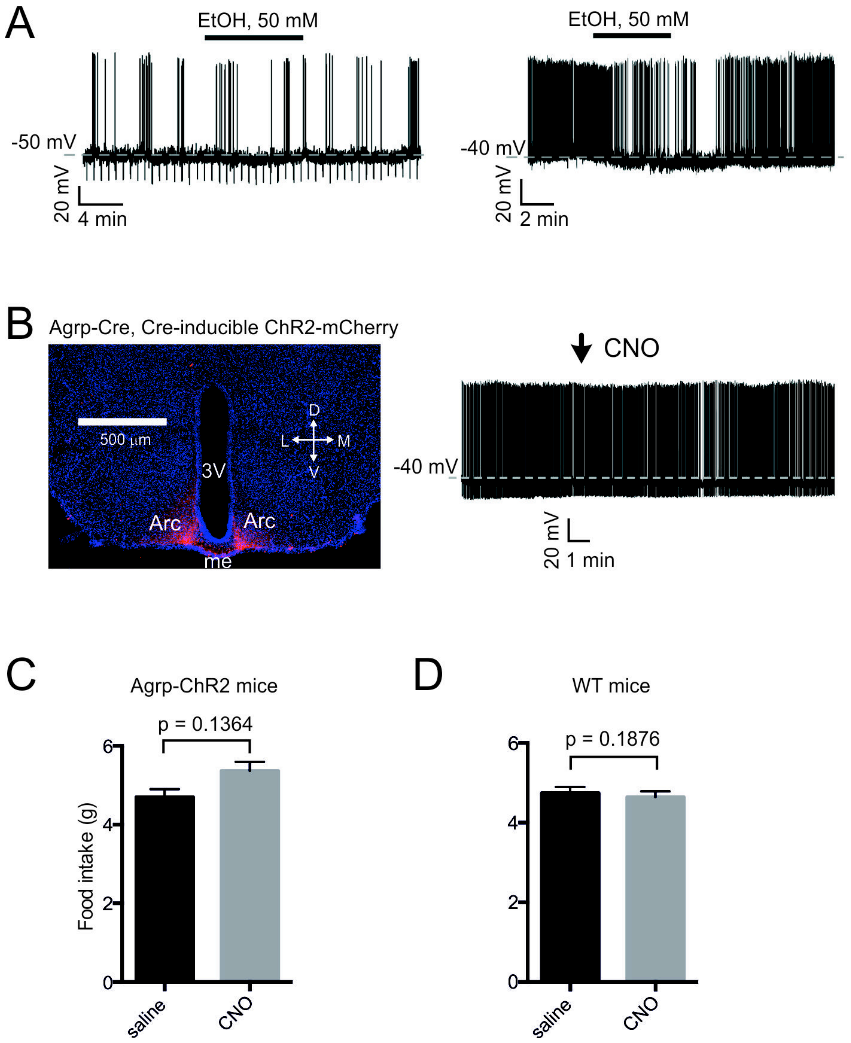

Supplementary Figure 1

A. Effects of 50 mM EtOH on membrane potential of non-Agrp neurons of the arcuate nucleus. Representative responses of 12 cells (5 cells, no effect; 7 cells; inhibited; overall change in firing rate = - 0.24±0.10 Hz, P = 0.0274 by paired t-test, t = 2.542, df = 11, n = 12 cells).

B. Effect of CNO on Agrp-Cre cells transfected with Cre-dependent ChR2-mCherry. Left, transgene targeting after stereotaxic injection. Right, representative response to 5 µM CNO of 3 cells.

C. Effect of CNO (5mg/kg i.p.) in mice expressing ChR2-mCherry in Agrp neurons (targeted as in B). Data are means±s.e.m. of n = 4 age-matched littermate pairs. P value is from a paired two-tailed t test: t = 2.022, df = 3.

D. Same as C, but in WT mice (n = 6 mouse pairs). P value is from a paired two-tailed t test: t = 1.526, df = 5.

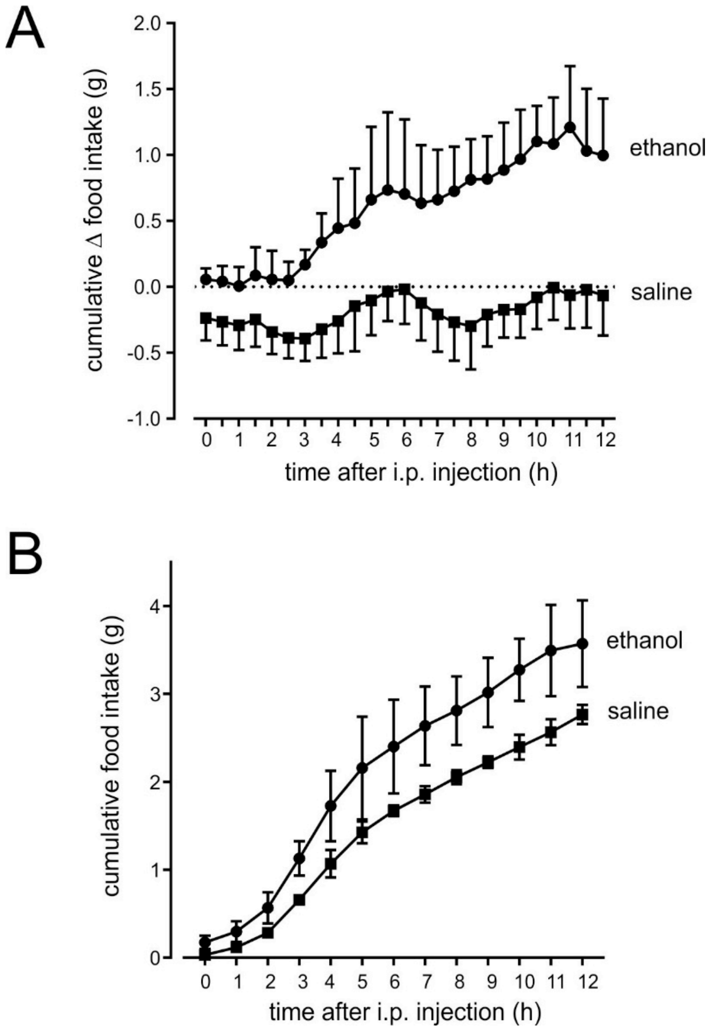

Supplementary Figure 2

Effect of EtOH on food intake explored using an alternative experimental design.
A single dose of EtOH (1g/kg), or equivalent volume of saline, was injected i.p. at 1600h, and food intake was measured in standard, unmodified TSE Phenomaster metabolic cages.

A. Time-course of EtOH-induced change in food intake (Δ food intake). For each mouse, during food intake on the day before the injections was subtracted from food intake on the day of injection, to generate the Δ food intake values at each 30 min. 4 male mice received a saline injection, and 4 male mice received an EtOH injection; the 8 mice were monitored concurrently in the metabolic cages. Two-way RM ANOVA, Interaction: $F (24, 144) = 1.717$, $P=0.0280$.

B. An alternative plot of the experiment described in A. This plot shows raw data (cumulative food intake) during the injection day, in EtOH or saline-injected mice, at each hour. Two-way RM ANOVA, Interaction: $F (12, 72) = 2.043$, $P=0.0322$.

Values are means+/- s.e.m.

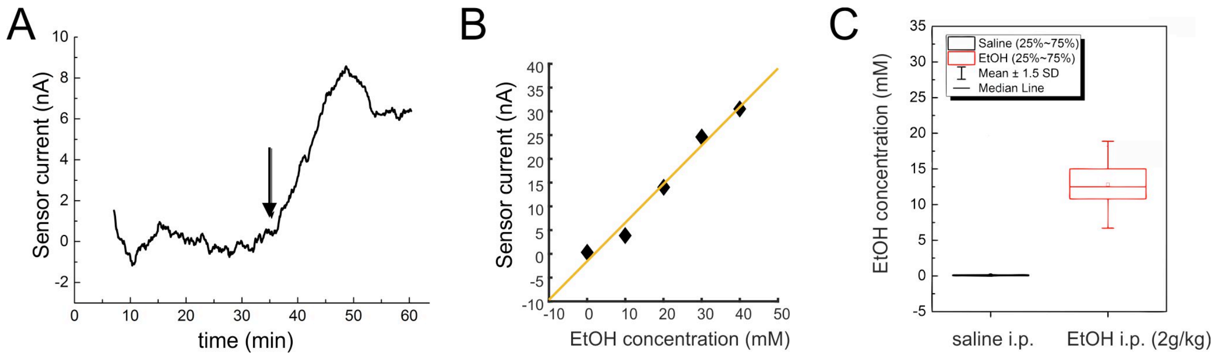

Supplementary Figure 3

Measuring deep brain ethanol levels with an electrochemical sensor.

A. Effect of an i.p. ethanol (EtOH) injection (2 g/kg, arrowed) on the signal of EtOH sensor implanted into the hypothalamic arcuate nucleus.

B. An example of calibration of the EtOH sensor electrode (performed individually for each electrode).

C. Summary of group data from experiments such as that shown in A; EtOH responses were obtained by calibration such as that shown in B; n = 3 mice in each group.

Ethanol sensor: Sensors were assembled based on a previously-developed design for ethanol detection in the brain (Rocchitta et al, Anal. Chem. 2012, 84: 7072-7079). Namely, 125 µm diameter platinum wires were coated with an approx. 1 µm thick PMMA-based insulation layer. A 500 µm wide circular insulator band was removed by means of a diamond blade Dremel, exposing Pt surface as a substrate for further modification. Ascorbic acid interference was blocked by electrodeposition of a thin poly-phenylenediamine layer (Kirwan et al, Sensors 2007, 7: 420-437) directly on the Pt surface. oPD was deposited by means of electropolymerization. Modified electrodes were rinsed in double distilled water (18 MΩ) and stored for further modifications. PEI and Glyc solution mix for enzyme stabilization was prepared and stored at 5°C. The enzyme solution was freshly prepared before the in vivo ethanol experiments. The sensor length was modified with alcohol oxidase and stabilizers by dip-evaporation during 10 alternative quick dips in the stabilization mix and the enzymes. For overall protection, a layer of PU was deposited by a single dip-evaporation step. The electrochemical cell was composed of a 3 electrode setup featuring a Pt (Ø = 1 mm) counter electrode, a Ag|AgCl/KClSAT reference electrode, and the ethanol-sensing working electrode. oPD electropolymerization was carried out on Pt at +0.7V vs REF for 20 minutes at room temperature and constant stirring (350 rpm).

In vivo detection: C57/BL6 mice (8–12 weeks old) were anaesthetized with urethane (2 g/kg i.p.), and head fixed using a head plate glued to the skull, and their body temperature was maintained at 37°C using a rectal probe and a heating blanket (FHC, ME, USA). An incision was made in the scalp and a small craniotomy was drilled and dura removed. Sensors were stereotaxically lowered into the hypothalamic arcuate nucleus (as described in the main Methods section), polarized to +0.7 V vs reference and kept until the exponential decay entered a linear phase (<0.1 nA/min). After recording the baseline signal, mice were given 2 g/kg ethanol i.p.

Chemicals: Chemicals were purchased from Sigma (Sigma-Aldrich, UK) except otherwise stated and used without further purification. 400 U mL^{-1} alcohol oxidase enzyme from Hansenula polymorpha (AOx, EC 1.1.3.13) was dispersed in PBS. Polyethyleneimine and glycerol solutions were obtained by diluting stock solutions in bidistilled water (50% w/v and 87% w/v respectively). 300 mM ortho-phenylenediamine monomer solution (oPD) was prepared in deoxygenated PBS. 1 % w/v polyurethane solution (PU) was obtained by dissolving PU blocks in tetrahydrofuran (THF). Two-part Methacrylate copolymer-based (PMMA, Paladur, France) material was supplied by Heraus Kulzer (UK); platinum wires (99,99% Pt, Ø = 125 µm) were purchased from Alfa Aesar (UK).
